# A prospective double-blinded study evaluating threshold doses of dietary allergens that trigger adverse food reactions in dogs, and time to flare after exposure

**DOI:** 10.3389/fvets.2026.1767167

**Published:** 2026-05-20

**Authors:** Laura Udraite Vovk, Laura Widorn, Georg Lehner, Ralf S. Mueller

**Affiliations:** 1 Center for Clinical Veterinary Medicine, Ludwig Maximilian University, Munich, Germany; 2 Kleintierpraxis Lehner, Fachpraxis für Dermatologie, Buch, Germany

**Keywords:** adverse food reactions, canine atopic dermatitis, dietary proteins, elimination diet trial, oral food challenge, threshold dose, time to flare

## Abstract

**Introduction:**

Adverse food reactions (AFR) are a common cause of chronic pruritus in dogs and often present as atopic dermatitis. However, the threshold dose of dietary protein required to trigger clinical signs of AFR, as well as the time to onset of those signs, remain poorly defined.

**Methods:**

This prospective, double-blinded study aimed to determine the approximate dose of food protein required to elicit clinical signs in dogs with previously confirmed AFRs and to assess the time to flare (TTF) following a single dietary protein provocation. Eleven dogs with confirmed AFR underwent 71 randomized oral food challenges (OFC) with seven individual protein sources in escalating doses for each protein over 7 days.

**Results:**

Clinical signs were observed in 35 challenges, most commonly between days 2 and 6, with a mean TTF of 4.1 days (range: 1–7 days). Reactions were mostly triggered by 20–30 g of food protein, with a mean dose of 21 g (range: 1–30 g). When adjusted for body weight, this corresponded to a median eliciting dose of approximately 0.86 g/kg (range: 0.06–2.5 g/kg).

**Discussion:**

While most dogs reacted to moderate-to-high protein exposure after repeated OFC, the variability in individual responses in both threshold dose and TTF highlights the need for further studies to refine diagnostic protocols and define clinically relevant threshold doses.

## Introduction

Canine atopic dermatitis (CAD) is one of the most common dermatological diseases in dogs (1, 2). Historically, CAD was considered a condition triggered exclusively by environmental allergens (3), while food allergy was classified as a separate disease (4, 5). However, due to overlapping clinical presentations and similarities in pathogenesis, it is now recognized that many dogs with food-induced hypersensitivity present with clinical signs of CAD and that food allergens can act as triggers for CAD flares (5–7). Adverse food reactions (AFRs) are broadly defined as abnormal clinical responses triggered by the ingestion of food or dietary ingredients and include both immunologically mediated reactions (food allergies) and non-immunologic responses, such as intolerances due to pharmacologic, toxic, metabolic, or idiosyncratic mechanisms (6). In the veterinary dermatology literature, however, the term AFR is commonly applied to describe food-induced hypersensitivity reactions and is often used interchangeably with “food allergy” (5). This terminology is followed accordingly in the present study. The reported prevalence of AFR in allergic dogs varies widely across studies, with an average of 29% (8), emphasizing the significant role of food allergens in canine hypersensitivity reactions.

Understanding the immunological mechanisms is essential for the interpretation of clinical responses and designing appropriate diagnostic protocols. This is particularly relevant in AFR, where the clinical presentation shows considerable diversity due to different immunological mechanisms, breed-specific patterns, and other influencing factors (9). While IgE-mediated type I hypersensitivity has been reported in dogs, it appears to occur infrequently (10). More commonly, dogs with cutaneous manifestations of AFR show a chronic pruritic phenotype consistent with delayed T-cell–mediated type IV hypersensitivity (11). In contrast, dogs with exclusively gastrointestinal AFR do not exhibit pruritus or skin lesions and may present with a more rapid onset of clinical signs after exposure. This predominance of delayed-type immune responses explains the gradual onset of clinical signs after exposure and provides the rationale for assessing the TTF in the present study.

Diagnosing food-induced CAD is challenging as it is clinically indistinguishable from its environmental counterpart, despite requiring different management (6, 7). Although a range of diagnostic methods has been studied, including intradermal, serum, salivary, hair, and gastroscopic testing, none have shown sufficient reliability (12, 13). Thus, an elimination diet trial (EDT) followed by dietary provocation remains the gold standard in the diagnostic workup of CAD. However, clinical improvement during EDT alone does not confirm AFR. Seasonal variation in environmental allergens and the use of symptomatic therapies, such as anti-inflammatory and antipruritic drugs, medicated shampoos, or essential fatty acids, can mimic a dietary response and confound interpretation (7, 14, 15). While it is often necessary to maintain symptomatic treatments in the beginning of the EDT to support patient comfort and owner compliance, this may interfere with diagnostic accuracy. Some medications, including oclacitinib and prednisolone, have been reported to shorten the required duration of the EDT (16, 17), though their potential to mask dietary effects must be considered. An improvement of clinical signs on EDT and deterioration upon controlled dietary rechallenge is therefore essential for a definitive AFR diagnosis. Failure to reintroduce the suspected allergen increases the risk of misdiagnosis and may lead to unnecessary dietary restrictions or inappropriate long-term treatment (7, 15).

The selection of an appropriate EDT must be adapted to each patient individually. Available options include a single novel protein, either home-cooked or commercial, or a hydrolyzed diet (HD) (14). However, commercial selected protein diets can be contaminated with trace amounts of other proteins not listed on the package (18–21). Defining the threshold for development of clinical signs is crucial for both accurate diagnosis and appropriate dietary formulation. Identifying a truly novel protein has become increasingly difficult, as many pets are exposed to a wide variety of commercial foods, treats, and table scraps (15). In addition, cross-reactivity between related and unrelated proteins may further complicate the choice (22, 23). These limitations have led to increasing concerns regarding the reliability of single novel-protein diets, and hydrolyzed products are therefore often preferred.

Conducting an EDT trial poses challenges for both veterinarians and pet owners. It requires considerable time and effort to explain how the diet works and why it is essential. Owners are often hesitant to comply, particularly when cooking is required, the dog refuses the new food, or when other family members, especially young children or elderly individuals, do not adhere to the dietary rules (15). These factors can all contribute to loss of compliance and owner adherence is a major factor in the success of the EDT. During allergy consultations, veterinarians often address diet, symptomatic treatment, disease pathogenesis, and short- and long-term management plans. This volume of information can easily overwhelm the client and further reduce compliance. As a result, HDs are often preferred for their convenience and improved owner adherence (15, 24).

The objective of this study was to determine the approximate threshold dose of major dietary protein allergens required to trigger clinical signs in dogs with confirmed AFR and to assess the TTF following an oral provocation with a single source. Defining these parameters may improve the selection of truly allergen-free elimination diets, clarify the clinical risk posed by trace allergen exposure, and help food manufacturers establish thresholds of clinically relevant labeling limits.

Despite the known importance of dietary provocation after EDT in confirming AFR, no veterinary studies have systematically quantified and clearly defined allergen threshold doses or characterized TTF after a single challenge in client-owned dogs.

Previous reports relied on open or single-blind provocations and focused on whether reactions occurred, not on dose–response relationships. To the authors’ knowledge, this is the first prospective, double-blinded study using controlled oral food challenges to evaluate both the minimal eliciting dose and time to relapse in naturally affected dogs. By addressing these gaps in the literature, this work aims to improve, standardize, and clarify diagnostic and dietary strategies for managing AFR in clinical practice.

## Materials and methods

### Study population

This was a prospective, double-blinded study conducted in client-owned dogs with a confirmed diagnosis of AFR, established by board-certified veterinary dermatologists based on an EDT and dietary rechallenge.

Dogs were eligible for inclusion if they had previously shown clinical improvement of their clinical signs on an EDT, followed by deterioration upon reintroduction of their original diet, and subsequent improvement when feeding the elimination diet again. At the time of enrollment, all dogs were clinically stable while maintained on a well-tolerated diet, defined as absence of clinically relevant pruritus or clinical lesions, no secondary skin infections, normal stool quality, and no ongoing gastrointestinal signs. The composition of individual elimination diets or otherwise well-tolerated diets and detailed histories of prior dietary exposure to specific protein sources were not standardized or systematically recorded, as this was not a predefined objective of the study. A total of 11 dogs were included (Labrador Retrievers *n* = 4, Boxers *n* = 3, and one each of Shih Tzu, Coonhound, Magyar Vizsla, and a mixed-breed dog), consisting of 6 males and 5 females, aged 1–12 years, with a body weight range of 7.5–38 kg and 71 individual OFC were performed. The study was approved by the institutional ethics committee under the number 149–29-10-2018, and informed owner consent was obtained prior to enrollment.

### Food provocations

On Day 0, a thorough baseline assessment was performed. This included completion of a detailed initial questionnaire (Supplementary material 1), clinical examination, and evaluation of skin lesions using the validated Canine Atopic Dermatitis Lesion Index (CADLI) (25) and pruritus using a validated Visual Analog Scale (PVAS) (26). If pruritus or lesions were present (CADLI or PVAS > 0), secondary infections were ruled out by cutaneous cytology.

Following the baseline assessment, each dog underwent seven sequential OFCs using protein powders derived from beef, fish, wheat, corn, pork, lamb, and chicken. The meat-derived protein powders were provided by Royal Canin (France) and are routinely employed by the manufacturer in the production of commercial diets. Additional independent purity or contamination testing besides routine quality control of the manufacturer in Royal Canin’s own laboratory was not performed by the authors within the scope of this study. The wheat and corn powders were commercially available food-grade products obtained from standard retail sources, reflecting ingredients commonly accessible to pet owners for home feeding. The order of the individual protein sources for each dog was randomized using a computer-generated sequence. Each OFC lasted up to 7 days and followed a fixed dose-escalation protocol: 1 g of the test protein was added to the diet on day 1, followed by an increase to 5 g on day 2, 10 g on day 3, and 30 g daily from days 4 to 7. The protein powders were pre-packaged in daily doses and clearly labeled from A to G for each protein and from 1 to 7 for the days of rechallenge when the individual bag should be fed.

In addition to absolute protein doses (g), eliciting doses were also expressed relative to body weight (g/kg) to account for differences in dog size.

Clinical deterioration was defined as the first appearance of at least one clinical sign consistent with an AFR flare, including an increase in pruritus by at least 2 points on the PVAS scale, the development of skin lesions, and/or gastrointestinal signs such as diarrhea or vomiting. When these signs occurred during a provocation, owners were instructed to immediately contact the veterinary dermatologist. The OFC was then discontinued, and the dog was maintained on its previously well-tolerated baseline diet until clinical signs resolved. If clinical signs persisted for more than 7 days, the dog was re-evaluated in the clinic to assess potential secondary infections. If an infection was diagnosed, appropriate treatment was initiated. A minimum seven-day washout period without increased pruritus or clinical signs was required before initiating the next OFC.

### Statistical analysis

Given the small sample size, data were analyzed descriptively. Continuous variables (e.g., threshold dose and TTF) were summarized using simple descriptive statistics (mean and range). Categorical variables were reported as counts and percentages. No formal hypothesis testing or model-based analyses were conducted. As key parameters required for formal sample size estimation (such as expected variability in threshold dose and TTF under a standardized challenge protocol) were not available at study initiation, no formal *a priori* power calculation was performed.

### Monitoring and scoring

Owners recorded daily pruritus scores in a diary throughout the OFC phase and completed a post-challenge questionnaire after each food challenge (Supplementary material 2). These data were used to document any changes in clinical signs, including the TTF, duration, and severity of pruritus, as well as any other dermatological or gastrointestinal symptoms. The collected information also identified the earliest clinical signs suggestive of a reaction and was used to estimate the time to onset of clinical signs following exposure to a single protein. Throughout the study, all dogs remained under the supervision of a board-certified veterinary dermatologist.

### Final assessment

After completing all seven OFCs, each dog was presented to the clinic again for a final evaluation and CADLI and PVAS scores were reassessed. The complete dataset was reviewed for the presence of positive reactions, defined by an increase of 2 points on the PVAS scale, or the presence of diarrhea, vomiting or skin lesions attributable to a specific protein, and the time to onset of clinical signs was documented.

## Results

**Figure 1:**
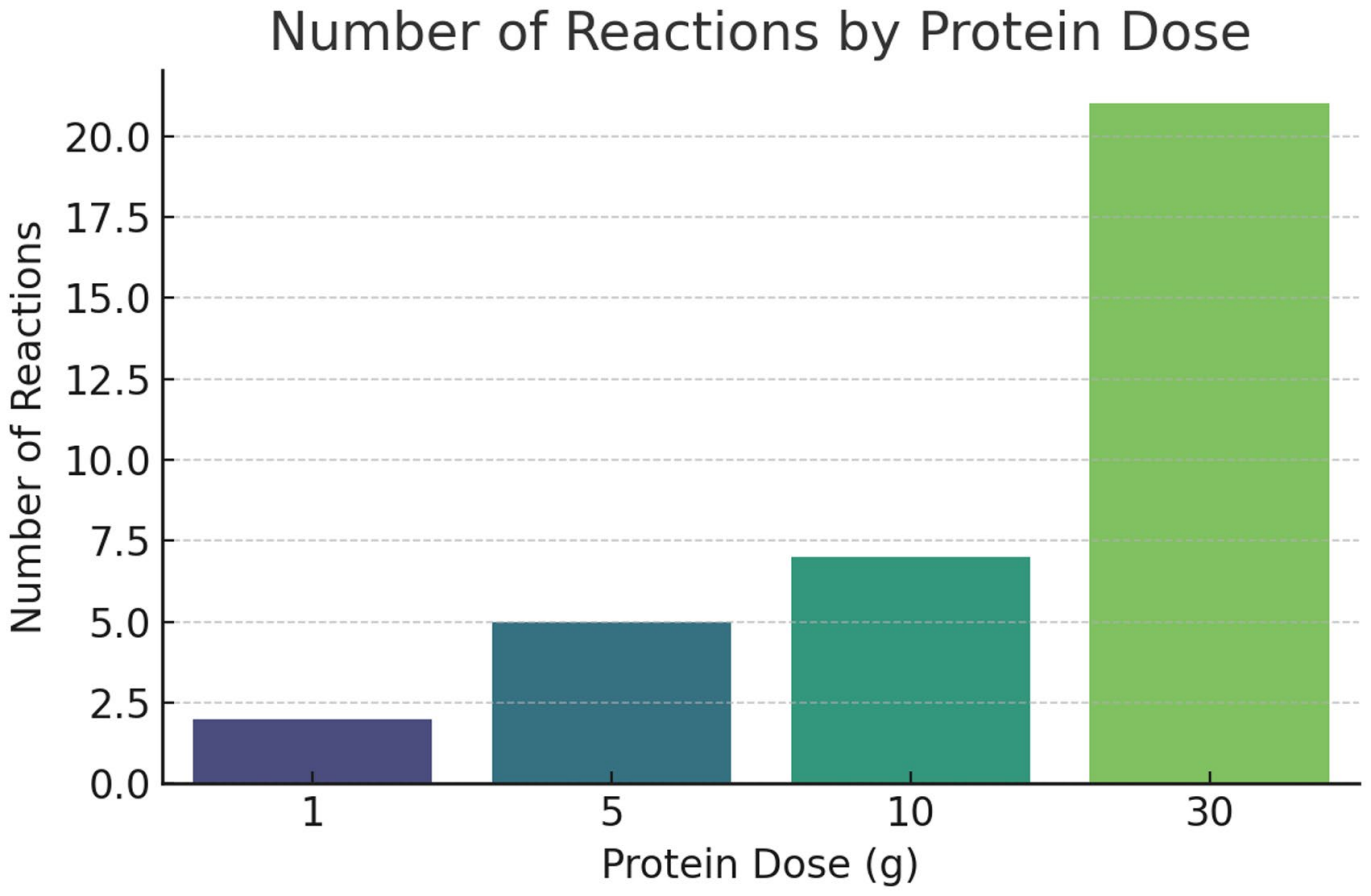
Distribution of reactions by protein dose.

**Figure 2:**
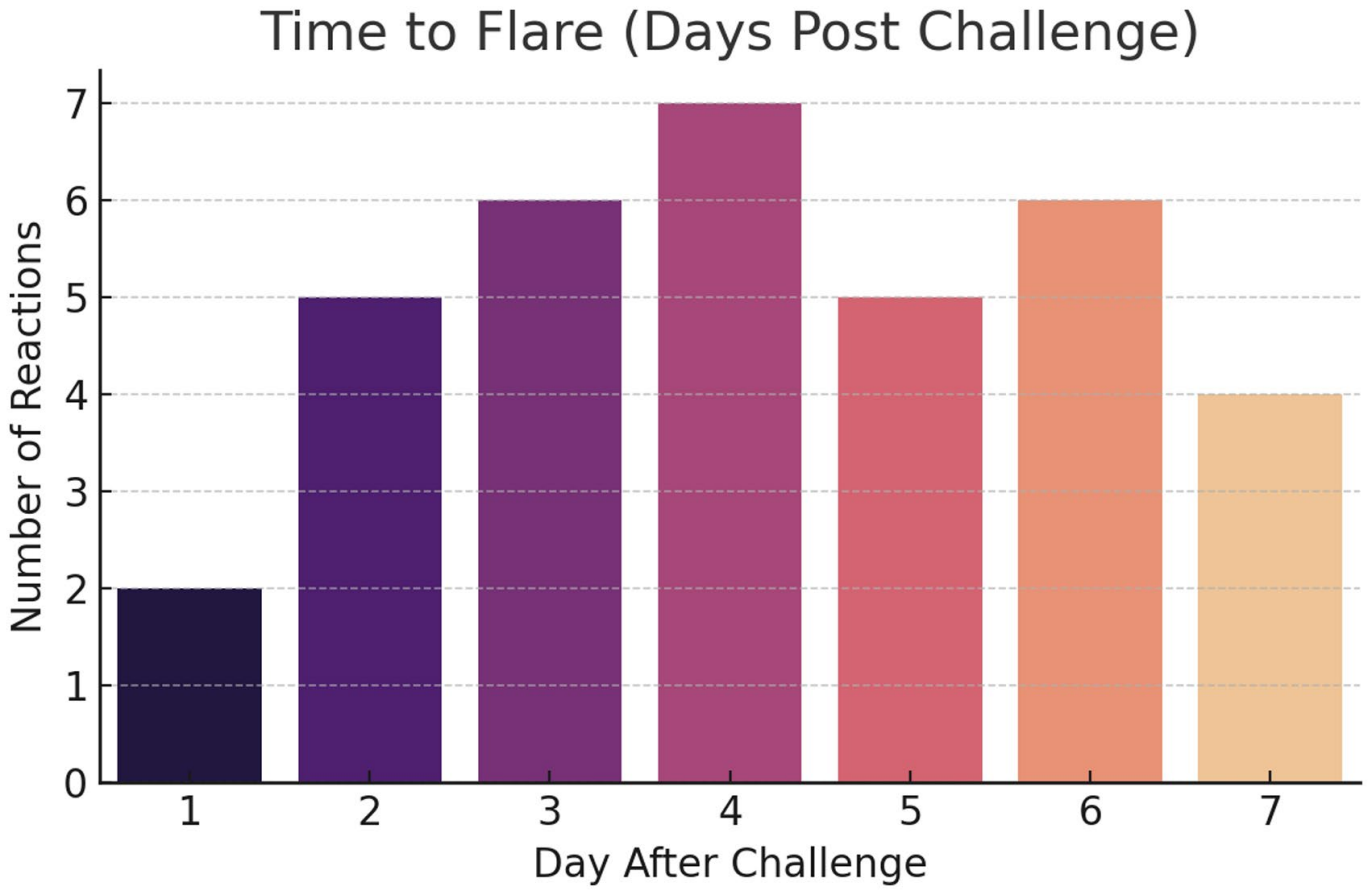
Time to flare (days) following oral food challenge.

**Figure 3:**
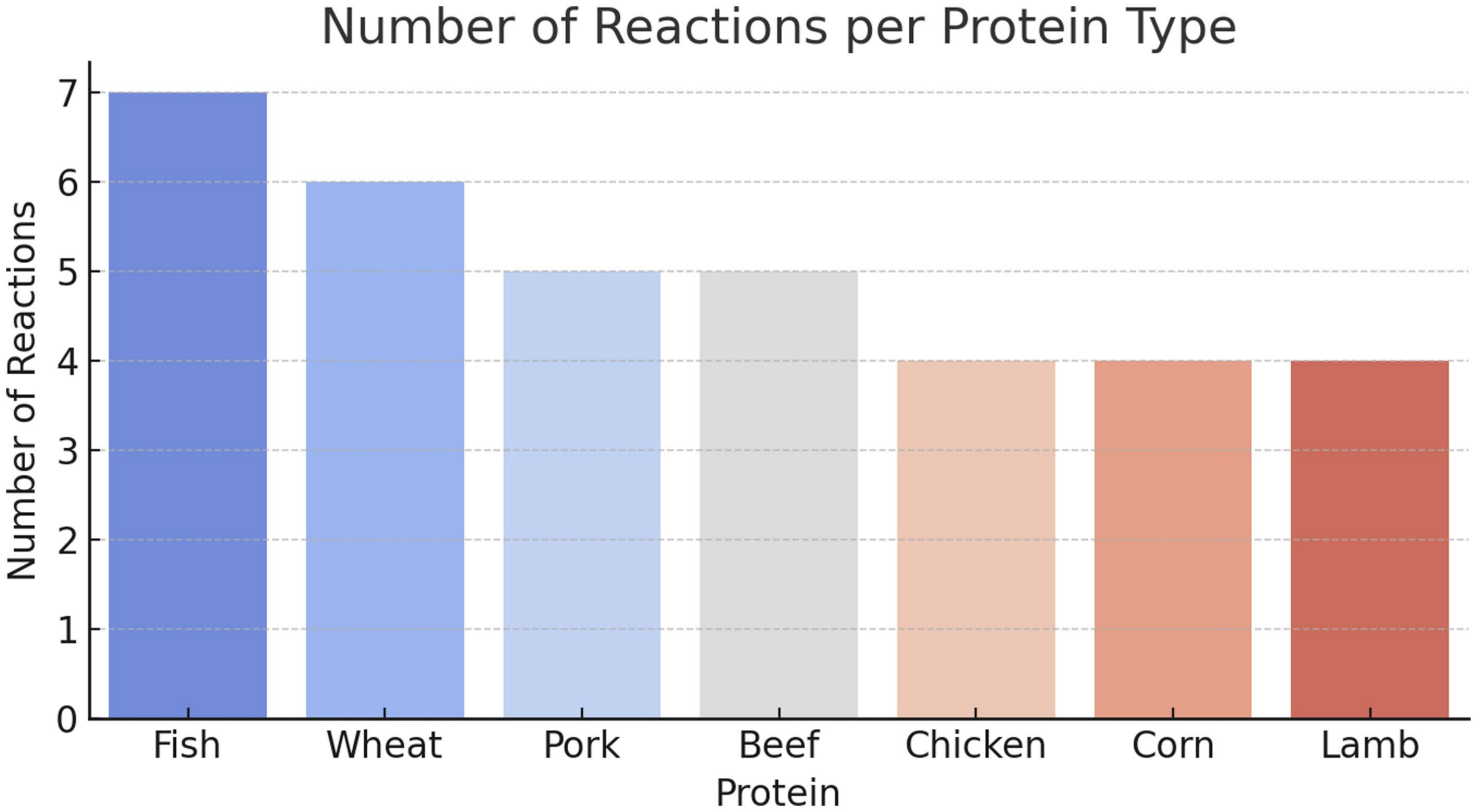
Number of reactions per dietary protein.

A total of 71 OFC were performed, 35 of which yielded positive results. Although 77 OFCs were initially planned (11 dogs challenged with seven protein sources), six challenges were not completed because two owners declined further provocations to avoid additional cutaneous and/or gastrointestinal flare-ups observed during first exposures. Ten of the eleven dogs showed more than one positive reaction, one dog did not react to any powdered allergen source. Positive reactions were observed at varying times, ranging from day 1 with 1 g of protein to day 7 with 30 g. Most of the reactions were observed at 30 g (*n* = 21, 60%), followed by 10 g (*n* = 7, 20%), 5 g (*n* = 5, 14.3%), and only 2 reactions (5.7%) were triggered by 1 g (Figure 1). For clarity, within the context of this study, a low protein dose refers to 1 g, moderate doses to 5–10 g, and a high dose to the highest challenge level (30 g) administered during oral food challenges. The mean response dose across all reactions was 20.8 g (range: 1–30 g). When adjusted for body weight, this corresponded to a median eliciting dose of approximately 0.86 g/kg (range: 0.06–2.5 g/kg). The number of days to reaction ranged from 1 to 7, with most reactions occurring between days 2 and 6 (Figure 2). The mean time to clinical deterioration (time to first clinical symptom, cutaneous or gastrointestinal) was 4.1 days (range: 1–7 days). Marked inter-individual variability was observed, with different dogs reacting at different protein doses (ranging from 1 to 30 g) and at different times following challenge (ranging from day 1 to day 7), and most dogs reacting to more than one protein source. Of the 10 dogs with positive OFC, 3 exhibited only cutaneous reactions, primarily pruritus, while 1 had only gastrointestinal signs, and 6 dogs experienced both. There were 7 reactions to fish, 6 to wheat, 5 each to beef and pork, and 4 each to chicken, corn, and lamb (Figure 3).

## Discussion

This prospective, double-blinded study investigated the protein threshold doses required to elicit clinical signs and the TTF in dogs with confirmed AFRs. Most dogs reacted to moderate-to-high protein exposures and clinical signs typically developed after several days, but there was considerable individual variability in both threshold dose and response timing.

### Threshold dose of protein allergens

Determining the exact threshold dose of food allergens required to trigger clinical signs in AFR in dogs is important for several reasons. First, threshold values help pet owners understand the risks of allergen exposure, whether a single spoonful of allergenic food is enough to trigger a reaction or if even trace amounts from kitchen surfaces pose a danger. This knowledge is also valuable for pet food manufacturers, helping guide precautionary allergen labeling and defining which trace levels are clinically relevant. Currently, studies employing polymerase chain reaction (PCR)-based techniques show that certain commercial monoprotein diets are contaminated with undeclared protein sources (18–21). PCR-based methods are commonly preferred because of their high sensitivity and species specificity, whereas ELISA assays may be affected by protein degradation and cross-reactivity. However, it remains unknown what quantitative threshold of such contaminating proteins is sufficient to trigger an allergic reaction in sensitized patients, and thus should be declared on the label, versus what level may be considered clinically irrelevant and tolerable. Currently, there are no published veterinary data defining precise allergen threshold doses in client-owned dogs. Only one published reference provided an exact quantitative challenge amount (200–500 g), which was used to induce clinical flares in experimentally sensitized laboratory dogs, rather than in client-owned patients, but a minimum threshold dose was not established (27). In a recent study, reactions to 1 teaspoon of protein were noted in 11% of food-allergic dogs, while others required larger amounts (28).

Results of our study suggest that although a small number of dogs may respond to minimal amounts of dietary protein (as low as 1 g), the majority require significantly larger quantities. As the Tinsley study used original diets with variable moisture and protein content (28), direct comparison to our use of dry powders is limited. For example, a teaspoon of chicken protein powder weighs approximately 3 g, whereas a teaspoon of cooked or raw chicken meat weighs around 5–6 g but contains a lower percentage of protein due to water and fat content. Despite these differences in moisture and matrix, the estimated amount of actual protein delivered per teaspoon is not expected to differ dramatically between these sources. In addition, protein processing methods such as drying, cooking, or hydrolysis may alter protein structure and epitope availability, potentially influencing allergenicity and further limiting direct comparisons between differently processed protein sources (29, 30). In contrast to the findings of Tinsley et al., where 11% of dogs reacted to a teaspoon-sized amount of the original diet, only 5.7% of dogs in our study showed a reaction to the smallest tested dose (1 g) of dry protein powder. Most dogs (60%) required approximately 20 g of dry protein powder to elicit a clinical flare, which corresponds to roughly 7 teaspoons of dry meat protein powder or approximately 11–13 teaspoons of cooked meat to achieve a comparable protein load (31). This represents a higher threshold than those triggering reactions in the Tinsley study and far exceeds typical thresholds seen in humans with food allergy (32, 33).

When eliciting doses were adjusted for body weight, no consistent relationship between dog size and allergen threshold was observed. The smallest dog in the study (7.5 kg) reacted at moderate doses (5–10 g) rather than at the lowest tested dose, whereas a medium-sized dog (17 kg) exhibited a reaction at the minimum dose administered (1 g). In addition, several larger dogs (28–38 kg) reacted at both moderate (5–10 g) and higher (30 g) absolute doses. Overall, there was substantial overlap in eliciting doses across dogs of different body sizes, indicating that inter-individual variability rather than body weight alone appeared to be the primary determinant of reaction thresholds.

In human allergology, the concept of eliciting doses (ELDs) is well established as a means of quantifying the minimal amount of an allergen required to induce a clinical reaction in allergic individuals (33, 34). ELDs are typically determined through controlled oral food challenges and are expressed in milligrams of allergenic protein. For example, ELD5 refers to the amount of allergen in milligrams that is expected to provoke a reaction in 5% of allergic individuals. This metric is particularly relevant in the context of IgE-mediated (type I hypersensitivity) reactions, which may result in life-threatening anaphylactic shock at very low allergen doses (35). To reduce this risk, food allergen thresholds and detection of these allergens in the food industry in human medicine are set accordingly low. Morisset et al. in 2003 demonstrated that, based on a standard intake of 100 g of food, a detection sensitivity of 10 parts per million (p.p.m) for egg, 24 p.p.m. for peanut, and 30 p.p.m. for milk protein would be required to protect 95% of allergic individuals from an allergy flare. Here, p.p.m. denotes the concentration of allergenic protein per kilogram of food and allows translation of eliciting doses into practical units applicable for labeling regulations and test sensitivity. As food-induced severe anaphylactic reactions due to masked allergens is an increasing problem in human medicine, contamination of manufactured products by unlabeled food allergens is a key concern for the food industry (32).

This illustrates how much lower the reactive doses are in humans, with reported thresholds of ELD5 less than 0.3 mg of hazelnut or 1.1 mg of milk, or ELD10 of 27 mg of fish (33, 36). The exceptionally low ELD values in humans reflect both the severity of potential reactions and the need for strict food safety regulation. In contrast, canine food allergies are very rarely associated with acute anaphylactic shock reactions (6, 10, 37). The predominant cutaneous clinical manifestation in dogs with AFR is chronic pruritus, which, while not life-threatening, can significantly reduce the quality of life for both the affected animal and its owner. Moreover, the underlying immunological mechanisms differ between species. While human food allergy studies primarily focus on type I IgE-mediated hypersensitivity reactions, cutaneous AFR in dogs, particularly food-induced AD, is believed to more commonly involve delayed, T-cell–mediated (type IV) hypersensitivity mechanisms, similar to those described for environmentally induced atopic dermatitis (11).

These immunopathological differences, along with the absence of systemic risk, may partially explain the lack of research on minimal eliciting doses in veterinary medicine. However, defining canine-specific triggering doses, even if considerably higher than human thresholds, remains clinically relevant. Quantification of such thresholds could better guide dietary recommendations, the interpretation of trace allergen contamination and may have implications for the design of elimination diets and food challenges in veterinary patients.

An important limitation of our study is that the dogs receiving the highest dose (30 g) were exposed to this amount for up to four consecutive days, whereas all lower doses were only administered for a single day, which could influence the observed threshold response.

### Time to flare after oral food challenges

An important aspect for planning the oral food challenges (OFCs) and performing studies on allergen threshold determination is understanding the time frame between exposure and onset of clinical signs, or TTF. Previous retrospective study reported that most dogs (60.9%) with CAD exhibited pruritus within 12 h after rechallenging and 23.9% within 3–6 h, while a small percentage exhibit delayed reaction, with one case showing clinical signs as late as day 10 (37). Occasionally, rechallenges occur as late as after 14 days of feeding the offending allergen (28, 38, 39). Accordingly, current recommendations suggest a rechallenge period of at least 2 weeks, although this may reduce owner compliance or lead to refusal to complete OFCs. If a shorter, more efficient re-challenge protocol could be established, it could significantly improve diagnostic adherence.

In our study, the mean TTF was 4.1 days, with most reactions (82.9%) occurring between days 2 and 6. Only two reactions (2/35, 5.7%) occurred on day 1, both in the same dog. This contrasts with the above-mentioned findings of Shimakura et al. in 2021, who reported that the majority of dogs reacted within 12 h and only a minority showed delayed responses. No consistent differences in time to flare were observed between individual protein sources, with reaction timing appearing to be primarily influenced by individual dog responses rather than by the specific protein challenged.

Considering our results, a monitoring period of at least 1 week after each dietary challenge appears necessary to reliably detect food-induced flare-ups in sensitized dogs. A longer observation window of up to 2 weeks, as recommended in previous studies and widely used as the gold standard, might be still more appropriate.

The flare pattern observed in our study differs from Shimakura’s findings in terms of timing, with reactions in our dogs occurring predominantly several days after the exposure rather than within hours. Importantly, although many reactions in the Shimakura study occurred within 12 h, they still fall within a delayed time frame compared with immediate IgE-mediated reactions typically described in human food allergy. These differences in reaction timing may reflect variations in study design, owner monitoring intensity, and dose escalation. Our results also differ from patterns typically reported in human medicine, where food-allergic reactions often occur immediately after re-exposure (35, 36). This discrepancy likely reflects underlying immunologic differences, as humans more commonly experience immediate (IgE-mediated) hypersensitivity, whereas dogs tend to develop delayed (T-cell–mediated) responses.

Delayed-type hypersensitivity reactions depend on antigen processing and lymphocyte activation, typically leading to slower onset of clinical signs compared to immediate IgE-mediated responses. This biological mechanism supports our observation of delayed flares and highlights why only short-term monitoring may miss relevant reactions. Furthermore, the poor correlation of IgE-based diagnostic tests with clinical outcomes, compared with the higher concordance of lymphocyte proliferation assays (12, 39), reinforces the role of cellular immunity in AFR pathogenesis.

### Protein distribution, cross-reactivity, and clinical implications

The dietary protein sources selected for OFC were chosen because they represent some of the most commonly used ingredients in commercial dog foods and as beef, chicken, wheat, lamb, and fish have been reported as the most frequent dietary triggers of AFRs in dogs across different geographic regions, including Europe, North America, and Australia (40).

No single protein predominated in causing flare-ups in our study. Reactions were distributed across various proteins, including fish (7 reactions), wheat (6), pork and beef (5 each), and chicken, corn, and lamb (4 each). This supports the concept that food hypersensitivity arises from aberrant immune responses during sensitization, rather than the intrinsic allergenicity of particular proteins (40). The immune system—not the protein itself—is responsible for the pathogenesis of hypersensitivity reactions. Sensitization may depend on prior exposure, meaning that the likelihood of a reaction is influenced by which food proteins an individual animal is or was commonly fed. As a result, the most frequently implicated allergens might vary regionally, depending on local feeding practices and ingredient availability. For example, lamb could be a more common allergen in Australia reflecting its widespread use in commercial diets. This highlights the importance of considering geographic and dietary context when evaluating suspected food allergens in clinical practice.

Furthermore, cross-reactivity plays a critical role in clinical manifestations. Structural similarities between related proteins (e.g., among animal meats or cereal grains), and in some cases even between unrelated sources, may lead to immune responses that appear to be directed at multiple distinct allergens. As a result, it is rarely possible to attribute a clinical reaction to a single defined allergen with certainty. This complexity underscores the importance of considering cross-reactions in both diagnostic and dietary strategies when managing adverse food reactions in dogs. It is also important to note that most published studies assessing allergen cross-reactivity in dogs are based on IgE measurements rather than T-cell reactivity (22). As the majority of AFRs are believed to be mediated by T-cells, the true extent and clinical relevance of cross-reactivity may differ from what is suggested by IgE-based data.

### Hydrolyzed diets and residual antigenicity

Hydrolyzed diets are widely used as first-line options in elimination trials because they minimize owner error and are typically produced using stringent manufacturing and quality control procedures, which reduce the risk of unintended protein contamination compared with selected-protein diets (19, 20, 24). However, their efficacy is not universal. Industrial hydrolysis primarily destroys conformational IgE-binding epitopes but may leave small linear peptide fragments (1–3 kDa) that can still be presented by canine MHC II molecules and activate T lymphocytes. Masuda et al. demonstrated that feather- and chicken-based hydrolysates induced T-cell proliferation in up to 39% of sensitized dogs, confirming that residual epitopes can persist despite hydrolysis (29). These mechanisms could explain occasional, typically milder relapses in dogs fed HDs derived from proteins to which they were previously sensitized. The likelihood of such T-cell-mediated reactions is expected to decrease with increasing degrees of hydrolysis (< 1 kDa) but is unlikely to be eliminated entirely. In addition to antigen reduction, HDs may exert microbiome-modulating effects that contribute to clinical improvement in some dogs with chronic enteropathies (41, 42). Alterations in the intestinal microbiome may influence mucosal barrier function and immune responses, potentially affecting susceptibility to AFRs (42). Disruption of intestinal barrier function and oral tolerance has been proposed as an important factor in AFRs (43), although their specific relevance to cutaneous manifestations in dogs is not well defined. Clinical responses may also be influenced by factors unrelated to antigenicity, including overall macronutrient composition, digestibility, and fiber content and distribution. Overall, HDs provide practical advantages in compliance and contamination control but cannot be assumed to be completely nonallergenic, a fact that should be considered when interpreting elimination or rechallenge outcomes.

### Compliance and diagnostic adherence

Compliance with elimination diets remains a major challenge in clinical practice. Veterinary dermatologists frequently encounter difficulties in maintaining strict adherence to dietary trials, with additional concerns such as cross-contact during food preparation—where even trace amounts of allergens (e.g., from shared utensils) may be sufficient to trigger a flare (44). The threshold for such cross-contact-induced reactions remains unknown, adding further uncertainty for both clinicians and pet owners. Although elimination diets initiate the diagnostic process, the rechallenge phase is essential to confirm AFR—but owners may hesitate to reintroduce allergens due to fear of flare-ups. By identifying approximate threshold doses that trigger clinical signs, our study aimed to improve understanding of how much allergen exposure is required to provoke a relapse. While definitive conclusions regarding cross-contact cannot be drawn, these data may help to understand if minimal cross-contact—such as trace residues on utensils or kitchen surfaces—could be associated with clinical flares in some dogs. Moreover, understanding the typical TTF may support the development of shorter, evidence-based rechallenge protocols, reducing the length and burden of diagnostic trials and thereby improving owner compliance.

As known from compliance research in other fields of veterinary medicine, providing more than five instructions per visit significantly reduces owner adherence (45). In routine dermatology practice, consultations often involve complex discussions covering elimination and provocation protocols, symptomatic treatment options (both topical and systemic), and short- and long-term management strategies. This volume of information can easily overwhelm clients, making strict compliance with elimination diets and the rechallenge phase even less likely.

### Study relevance and next steps

Our study aimed to explore whether the duration of the rechallenge diet trial phase could be shortened and whether threshold dosages might be identified to help guide elimination diet choices. However, while our findings provide estimates of mean thresholds and flare timing, individual variation prevented reliable reduction of monitoring periods or universal thresholds. Further studies are needed to address these questions.

### Limitations

This study has several limitations that should be considered when designing future investigations.

First, the number of tested dogs was small. Although the original plan was to conduct a multicenter study involving more dogs, only 11 patients were enrolled over the study period. This highlights the challenges of multicenter trials in veterinary dermatology, particularly the difficulty in persuading owners to consent to rechallenges when their previously pruritic dogs are in full remission. All dogs with a confirmed diagnosis of AFR that were either evaluated during the study period or previously followed at the participating centers were considered for inclusion, however, enrollment depended on owner consent and the ability to comply with the rechallenge protocol. The exact number of dogs screened but not enrolled was not prospectively recorded. Non-participation was most related to owner reluctance to proceed with dietary rechallenges in clinically stable dogs in the authors’ institutions and the lack of recruitment in other study centers. Because of the small sample size and the repeated-measures design with multiple challenges per dog, results were primarily analyzed descriptively to avoid overinterpretation of underpowered inferential statistics. As key parameters required for formal sample size estimation (such as expected variability in threshold dose and time-to-flare under a standardized challenge protocol) which were among the objectives of the present study, were not available at study initiation, no formal *a priori* power calculation was performed.

Second, due to recruitment constraints, the rechallenge period was limited to 7 days, rather than the full 14-day duration typically recommended. This decision aimed to improve owner compliance by minimizing the overall burden of participation. However, this limits the interpretation of our TTF data, as we cannot exclude the possibility that some dogs would have reacted later. Indeed, one patient in our study flared as late as Day 7, suggesting that, as reported before, longer monitoring periods may be necessary in some cases to fully capture delayed hypersensitivity responses.

Third, the rapid dose-escalation protocol was selected based on the hypothesis that true allergic reactions often occur at low protein exposures and manifest quickly (37), making slower increases (potentially reducing owner compliance) unnecessary. While this design was practical, it may have influenced the observed thresholds, as higher doses were repeated for several days while lower ones were only given once.

Fourth, the study protocol initially planned to repeat an OFC with the protein to which each dog had reacted, at the end of that dog’s complete series of provocations, to further confirm individual reactions. In practice, this confirmation challenge could only be performed in a limited number of dogs. Once a clear cutaneous and/or gastrointestinal flare had been observed, most owners declined additional provocations to avoid intentionally inducing further clinical signs. As a result, repeat challenges with the offending protein were not performed consistently across all dogs and were therefore not included in the outcome analysis.

Fifth, we did not include a formal placebo group. In a recent study, two of 10 dogs reacted to a placebo—the elimination diet blended with water, without added challenge proteins—indicating that placebo responses can occur (7). Such reactions may reflect the owner’s expectations or altered perception of pruritus rather than true immunologic responses. Without this comparison, we cannot fully rule out that some mild or ambiguous responses in our allergic cohort represented false-positive events attributable to owner bias or nonspecific factors rather than antigen-specific hypersensitivity.

Sixth, we did not capture the exact time to flare following Day-1 provocation. Owners were not instructed to record whether clinical signs appeared within minutes, within 3–6 h, or later, limiting our ability to distinguish between immediate type I hypersensitivity and delayed type IV responses. Future studies should aim to document the time course more precisely, either through in-clinic provocations under observation or at-home challenges performed when continuous owner monitoring is possible. Nonetheless, subtle signs such as faint erythema or transient licking may still be overlooked at home, whereas low-grade pruritus could be masked in a clinic setting due to stress.

Seventh, the protocol involved stepwise increases in protein quantity, which introduced an additional layer of complexity. It remains unclear whether clinical signs in some dogs were triggered by the amount of protein ingested, the duration of exposure, or a combination of both. Importantly, the same absolute quantity may not carry equal biological relevance across dogs of varying body sizes. For example, a 1 g portion represents a much higher per-kilogram dose for a small-breed dog such as a Yorkshire Terrier than for a large-breed Labrador Retriever. Without dose standardization relative to body weight, individual thresholds may be underestimated or overestimated, particularly in small dogs.

## Conclusion

This study provides novel insights into the threshold protein doses and time to flare in dogs with confirmed AFRs. While a small proportion of dogs reacted to as little as 1 g of dietary protein, most required larger quantities (mean: 20.8 g, median: 0.86 g/kg), and time to flare was often delayed, with a mean of 4.1 days. These findings suggest that food challenges should be monitored for at least 7 days, and possibly up to 14, to capture delayed reactions. Despite higher allergen thresholds in dogs compared to humans, quantifying these values remains critical for informing dietary strategies, labeling, and diagnostic accuracy in canine AFR.

Further multicenter studies with standardized, weight-based dosing and placebo controls are needed to refine thresholds and protocols for clinical use.

## Data Availability

The raw data supporting the conclusions of this article will be made available by the authors, without undue reservation.
