# Supplementary material for: A prospective double-blinded study evaluating threshold doses of dietary allergens that trigger adverse food reactions in dogs, and time to flare after exposure
Source: Front Vet Sci. 2026 May 20;13:1767167. doi: 10.3389/fvets.2026.1767167 (PMC13229624; doi:10.3389/fvets.2026.1767167)
Supplement: Supplementary file 1 [file Supplementary_file_1.docx]

**Supplementary Material 1**: **Initial questionnaire - Food study**

**General information**

Practice: ........................................................................ Country: .................................................

Owner name: ………………………....................................... Date: ......................

Pet name: ……………..…............ Breed: ………………………....................................... Age: ……......

***Gender***: o Male o Female o Castrated

***Environment***: o Mainly indoor o Mainly outdoor o Both in- and outdoor

***Other animals***: o No o Yes Specify: ..........................................................

***Life style***: o Free access to outdoors

o Walked once daily o Walked twice daily o Walked three times daily

o Vigorous excercise daily o Working dog Specify: .......................................................................

***Diet***: o Dry food o Canned food o Home-prepared food

Specify: ....................................................................................................................................................

Treats: o No o Yes Specify: ....................................................................................

Supplements: o No o Yes Specify: .......................................................................

***Previous problems***:

Skin problems: o No o Yes Specify: .......................................................................

GI problems: o No o Yes Specify: .......................................................................

Other problems: o No o Yes Specify: .......................................................................

**Information about previous tests & drugs**

***Flea control***

Specify: ......................................................................................................................................

***Drugs that helped:***

o Glucocorticoids o Cyclosporine o Oclaticinib o Lokivetmab

o Antibiotics o Antimycotics o NSAIAs o Antihistamines

o Shampooos o Other Specify: ........................................................................................

***Drugs that did not help:***

o Glucocorticoids o Cyclosporine o Oclaticinib o Lokivetmab

o Antibiotics o Antimycotics o NSAIAs o Antihistamines

o Shampooos o Other Specify: ......................................................................................

***Tests performed:***

CBC/Biochem: o No o Yes Specify: ..........................................................

Thyroid testing: o No o Yes Specify: ..........................................................

Cytology: o No o Yes Specify: ..........................................................

Skin scrapings: o No o Yes Specify: ..........................................................

Biopsy: o No o Yes Specify: ..........................................................

Allergy testing: o No o Yes Specify: ..........................................................

**Information about skin problems**

Start of skin problems: .............................

***Pruritus***: o No o Yes

o Pinnae o Ear canals o Muzzle o Periocular o Neck

o Lateral chest o Ventral chest o Axillae o Ventrum o Dorsum

o Front paws o Hind paws o Hocks o Carpi o Perianal area

***Lesions***: o No o Yes

o Redness o Bumps o Pustules o Crusts o Scales

o Pinnae o Ear canals o Muzzle o Periocular o Neck

o Lateral chest o Ventral chest o Axillae o Ventrum o Dorsum

o Front paws o Hind paws o Hocks o Carpi o Perianal area

***Saisonality***

o Strictly seasonal o Perennial o All year round but seasonally worse

Worse in: o Spring o Summer o Fall o Winter

What was seen first initially? o Lesions o Itching

**Gastrointestinal information**

On average, how often does your dog have belly pain, appear to have stomach cramping or an upset stomach?

o Multiple times per day o Daily o A few times per week o A few times monthly

o A few times per year

On average, how often does your dog strain to defecate or squat for long periods of time with no poop coming out?

o Multiple times per day o Daily o A few times per week o A few times monthly

o A few times per year

On average, how often does your dog defecate every day?

o Once daily o Twice daily o Three times daily o Four times daily

o More than four times daily

On average, how often does your dog have flatulence?

o Multiple times per day o Daily o A few times per week o Rarely o Never

Does your dog eat grass?

o My dog eats grass only to vomit o My dog eats grass but normally does not vomit thereafter

o My dog eats grass daily o My dog eats grass weekly

o My dog eats grass monthly o My dog eats grass very rarely

On average, how often does your dog regurgitate? This is when food (digested/nondigested) or water comes up with no stomach contractions. The food/water just comes out.

o Multiple times per day o Daily o A few times per week o A few times monthly

o A few times per year

On average, how often does your dog vomit?

o Multiple times per day o Daily o A few times per week o A few times monthly

o A few times per year

On average, how often does your dog scoot his or her bottom on the ground?

o Multiple times per day o Daily o A few times per week o A few times monthly

o A few times per year

Has your dog ever been treated by a veterinarian for an anal gland infection, rupture or abscess?

o Yes o No

If your pet needs its anal glands expressed regularly, how often is that needed?

o A few times per week o A few times per month o A few times per year
