# Supplementary material for: A prospective double-blinded study evaluating threshold doses of dietary allergens that trigger adverse food reactions in dogs, and time to flare after exposure
Source: Front Vet Sci. 2026 May 20;13:1767167. doi: 10.3389/fvets.2026.1767167 (PMC13229624; doi:10.3389/fvets.2026.1767167)
Supplement: Supplementary file 2 [file Supplementary_file_2.docx]

**Supplementary Material 2: Recheck questionnaire - Food study**

**General information**

Practice: ........................................................................ Country: .................................................

Owner name: ………………………....................................... Date: ......................

Pet name: ……………..…............ Breed: ……………………….......................... Age: ……......

***Diet***: o Commercial o Home-prepared diet o Rechallenge

Specify: …………………………………………………………………………………………………………….

***Flea control***

Specify: …………………………………………………………………………………………………………….

***Concurrent drugs:***

o Glucocorticoids o Cyclosporine o Oclaticinib o Lokivetmab

o Antibiotics o Antimycotics o NSAIAs o Antihistamines

o Shampooos o Other Specify: .............................................................................

***Pruritus***: o No o Yes

o Pinnae o Ear canals o Muzzle o Periocular o Neck

o Lateral chest o Ventral chest o Axillae o Ventrum o Dorsum

o Front paws o Hind paws o Hocks o Carpi o Perianal area

***Lesions***: o No o Yes

o Redness o Bumps o Pustules o Crusts o Scales

o Pinnae o Ear canals o Muzzle o Periocular o Neck

o Lateral chest o Ventral chest o Axillae o Ventrum o Dorsum

o Front paws o Hind paws o Hocks o Carpi o Perianal area

What was seen first initially? o Lesions o Itching

**Gastrointestinal information**

How often does your dog have belly pain, appear to have stomach cramping or an upset stomach now?

o Multiple times per day o Daily o A few times per week o A few times monthly

o A few times per year

How often does your dog strain to defecate or squat for long periods of time with no poop coming out now?

o Multiple times per day o Daily o A few times per week o A few times monthly

o A few times per year

How often does your dog defecate every day the last couple of days?

o Once daily o Twice daily o Three times daily o Four times daily

o More than four times daily

How often does your dog have flatulence now?

o Multiple times per day o Daily o A few times per week o Rarely

o Never

Does your dog eat grass now?

o My dog eats grass only to vomit o My dog eats grass but normally does not vomit thereafter

o My dog eats grass daily o My dog eats grass weekly

o My dog eats grass monthly o My dog eats grass very rarely

How often does your dog regurgitate now?

o Multiple times per day o Daily o A few times per week o A few times monthly

o A few times per year

How often does your dog vomit now?

o Multiple times per day o Daily o A few times per week o A few times monthly

o A few times per year

How often does your dog scoot his or her bottom on the ground now?

o Multiple times per day o Daily o A few times per week o A few times monthly

o A few times per year
